# Supplementary material for: Complete bio-degradation of poly(butylene adipate-co-terephthalate) via engineered cutinases
Source: Nat Commun. 2023 Mar 24;14:1645. doi: 10.1038/s41467-023-37374-3 (PMC10039075; doi:10.1038/s41467-023-37374-3)
Supplement: Supplementary file 1 — Supplementary Information [file 41467_2023_37374_MOESM1_ESM.pdf]

Supplementary Information

Complete bio-degradation of poly(butylene adipate-co-terephthalate) via engineered cutinases

Yu Yang[1,4], Jian Min[1,4], Ting Xue[1,4], Pengcheng Jiang[1], Xin Liu[1], Rouming Peng[1], Jian-Wen Huang[1], Yingying Qu[1], Xian Li[1], Ning Ma[2], Fang-Chang Tsai[2], Longhai Dai[1], Qi Zhang[3], Yingle Liu[3]*, Chun-Chi Chen[1]*, and Rey-Ting Guo[1]*

[1]State Key Laboratory of Biocatalysis and Enzyme Engineering, Hubei Hongshan Laboratory, Hubei Collaborative Innovation Center for Green Transformation of Bio-Resources, Hubei Key Laboratory of Industrial Biotechnology, School of Life Sciences, Hubei University, Wuhan 430062, PR China

[2]Hubei Key Laboratory of Polymer Materials, Key Laboratory for the Green Preparation and Application of Functional Materials (Ministry of Education), Hubei Collaborative Innovation Center for Advanced Organic Chemical Materials, School of Materials Science and Engineering, Hubei University, Wuhan 430062, PR China

[3]State Key Laboratory of Virology, College of Life Sciences, Wuhan University, Wuhan 430072, PR China

[4]These authors contributed equally: Yu Yang, Jian Min, and Ting Xue

*Corresponding authors. E-mail addresses: mvlwu@whu.edu.cn (Y. Liu), ccckate0722@hubu.edu.cn (C.-C. Chen), guoreyting@hubu.edu.cn (R.-T. Guo).

Supplementary Table 1. Summary of activity of enzyme-mediated PBAT hydrolytic activity.

Enzyme	Classification	Source	Yield (mg/L)	Activity (mol/mol)*	PBAT	Condition	Literature
PfL1	Lipase	*Pelosinus fermentans*	-	~ 30<br>~ 130	Film<br>Milled	50 ºC, 72 h	(AMB, 2016)[1]
PpEst	Esterase	*Pseudomonas pseudoalcaligenes*	-	~ 8<br>~ 70	Film<br>Milled	65 ºC, 72 h	(AMB, 2017)[2]
Cbotu_EstB	Esterase	*Clostridium botulinum*	-	< 30	Not specified	37 ºC, 72 h	(BB, 2016)[3]
*Tf*Cut	Cutinase	*Thermobifida fusca*	13.5	5198	Film	70 ºC, 48 h	This study
*Is*PETase	Cutinase	*Ideonella sakaiensis*	5.1	4868	Film	30 ºC, 48 h	This study
*Pb*PL	Cutinase	*Polyangium brachysporum*	1.1	UD	Film	30 ºC, 48 h	This study
*Bur*PL	Cutinase	*Burkholderiales* bacterium	1.5	3208	Film	35 ºC, 48 h	This study
*Tc*Cut	Cutinase	*Thermobifida cellulosilytica*	16.1	5361	Film	65 ºC, 48 h	This study

*The activity is the quantitation of products containing TPA and BTa (mol) per mol enzymes. UD, undetectable.

Supplementary Table 2. Representative degradation products and their theoretical mass in a negative ion ($M - H^-$) mode.

PBAT hydrolytic products	Mass to charge Ratio (m/z)	Present in this study
Products containing one TPA		
TPA (or Ta)*	165.0	**1**
BTa	237.1	**2**
BTaB	309.1	
ABTa	365.1	**3**
ABTaB	437.2	possible for **5**
ABTaBA	565.2	possible for **6**
BABTa	437.2	possible for **5**
BABTaB	509.3	
BABTaBA	637.3	
BABTaBAB	709.4	
ABABTa	565.2	possible for **6**
Products containing two TPAs		
TaBTa	385.1	**4**
BTaBTa	457.2	
ABTaBTaB	657.3	
BTaBTaB	529.2	

*TPA or Ta, terephthalic acid; B, 1,4-butanediol; A, adipic acid

Supplementary Table 3. Time course of TPA and BTa production by WT and *Tf*Cut-DM. The amounts of TPA and BTa are calibrated with the counterpart standard samples.

Time (h)	TPA (μM)			BTa (μM)		
	WT *Tf*Cut	*Tf*Cut-DM	Fold	WT *Tf*Cut	*Tf*Cut-DM	Fold
2	4.3 ± 1	61.1 ± 6.1	14.22	306.6 ± 27.5	413.5 ± 31.1	1.35
4	52.2 ± 5.2	174.5 ± 29	3.34	710.9 ± 63.8	763 ± 72.6	1.07
8	114.1 ± 15.1	442.8 ± 18.8	3.88	978.8 ± 30.3	1085.9 ± 47.2	1.11
12	309.8 ± 55.1	895.5 ± 72.9	2.89	1203.3 ± 71.6	1165 ± 141.9	0.97
24	628.4 ± 116.6	1568.6 ± 41.4	2.5	1192.5 ± 77.5	575.6 ± 141.7	0.48
36	852.2 ± 50.5	1697.5 ± 244	1.99	1312.5 ± 20.4	198.6 ± 60.3	0.15
48	705.3 ± 52.4	1748.2 ± 19.4	2.48	1114.1 ± 67.2	24.6 ± 2.1	0.02

Supplementary Table 4. Data collection and refinement statistics for crystal structures

	*Tf*Cut_DM_ Native	*Tf*Cut_DM_ S170A	*Tf*Cut_DM_ S170A_ MHET	*Tf*Cut_WT_ S170A	*Tf*Cut_WT_ S170A_ MHET	*Is*PETase_ S160A MHET
**PDB code**	7XTR	7XTS	7XTT	7XTU	7XTV	7XTW
Data collection						
Wavelength	1.34138	1.34138	1.34138	1.34138	1.34138	1.34138
Space group	$P12_11$	$P12_11$	$P12_11$	$P3_121$	$P3_121$	$P2_12_12_1$
Unit cell						
a, b, c (Å)	65.7, 42.0, 80.8	66.0, 43.0, 80.4	65.8, 41.9, 80.9	91.4, 91.4, 247.7	91.5, 91.5, 247.6	50.8, 50.9, 84.1
α, β, γ (°)	90, 92.6, 90	90, 92.4, 90	90, 92.1, 90	90, 90, 120	90, 90, 120	90, 90, 90
Resolution (Å)	37.2 – 2.20 (2.23 – 2.20)[a]	37.9 – 2.21 (2.24 – 2.21)	32.6 – 1.82 (1.85 – 1.82)	40.0 – 2.43 (2.47 – 2.43)	45.7 – 2.31 (2.35 – 2.31)	36.0 – 1.91 (1.94 – 1.91)
No. of observed reflections	21932 (860)	22833 (882)	39820 (1821)	46206 (2221)	53587 (2675)	17534 (762)
Redundancy	4.2 (3.0)	4.8 (3.0)	4.4 (3.2)	9.4 (4.5)	5.2 (3.0)	6.1 (5.5)
Completeness (%)	96.5 (95.9)	99.4 (99.4)	99.6 (98.1)	99.9 (99.8)	99.6 (99.4)	99.8 (98.3)
Average I/σ (I)	16.1 (8.5)	8.5 (4.4)	28.6 (9.0)	10.2 (2.1)	15.0 (4.7)	22.9 (9.5)
R_{merge} (%)[b]	6.4 (9.9)	12.0 (24.3)	3.4 (10.5)	15.9 (51.3)	6.9 (16.0)	5.3 (14.2)
Refinement[c]						
R_{work}	17.1	17.9	13.6	17.5	16.6	13.6
R_{free}	23.3	23.7	17.3	22.6	21.9	14.9
r.m.s.d. bonds (Å)[d]	0.007	0.007	0.006	0.009	0.007	0.007
r.m.s.d. angles (°)	0.868	0.927	0.853	0.978	0.907	0.940
Ramachandran statistics[e]						
Most favored (%)	97.2	96.9	97.7	96.5	96.6	98.1
Allowed (%)	2.8	3.1	2.3	3.5	3.4	1.9
Outliers (%)	0	0	0	0	0	0
Average B-factor (Å^2)						
Protein/atoms	15.8 / 3919	21.3 / 3974	10.5 / 4010	18.6 / 7976	16.5 / 7976	10.9 / 1922
Water/atoms	21.4 / 338	26.0 / 345	22.1 / 683	25.2 / 817	23.3 / 867	24.7 / 253
MHET/atoms	-	-	19.5 / 15	-	23.9 / 30	24.6 / 15
Other Ligands /atoms	25.5 / 23	19.8 / 2	20.0 / 19	28.7 / 96	30.0 / 80	37.8 / 45

a Values in parentheses are for the highest resolution shell.

b $R_{merge} = \Sigma_{hkl}\Sigma i|I_i(hkl) - \langle I(hkl)\rangle| / \Sigma_{hkl}\Sigma_i I_i(hkl)$, in which the sum is over all the *i* measured reflections with equivalent miller indices *hkl*; $\langle I(hkl)\rangle$ is the averaged intensity of these *i* reflections, and the grand sum is over all measured reflections in the data set.

c All positive reflections were used in the refinement.

d According to Engh and Huber[4].

e Calculated by using MolProbity[5].

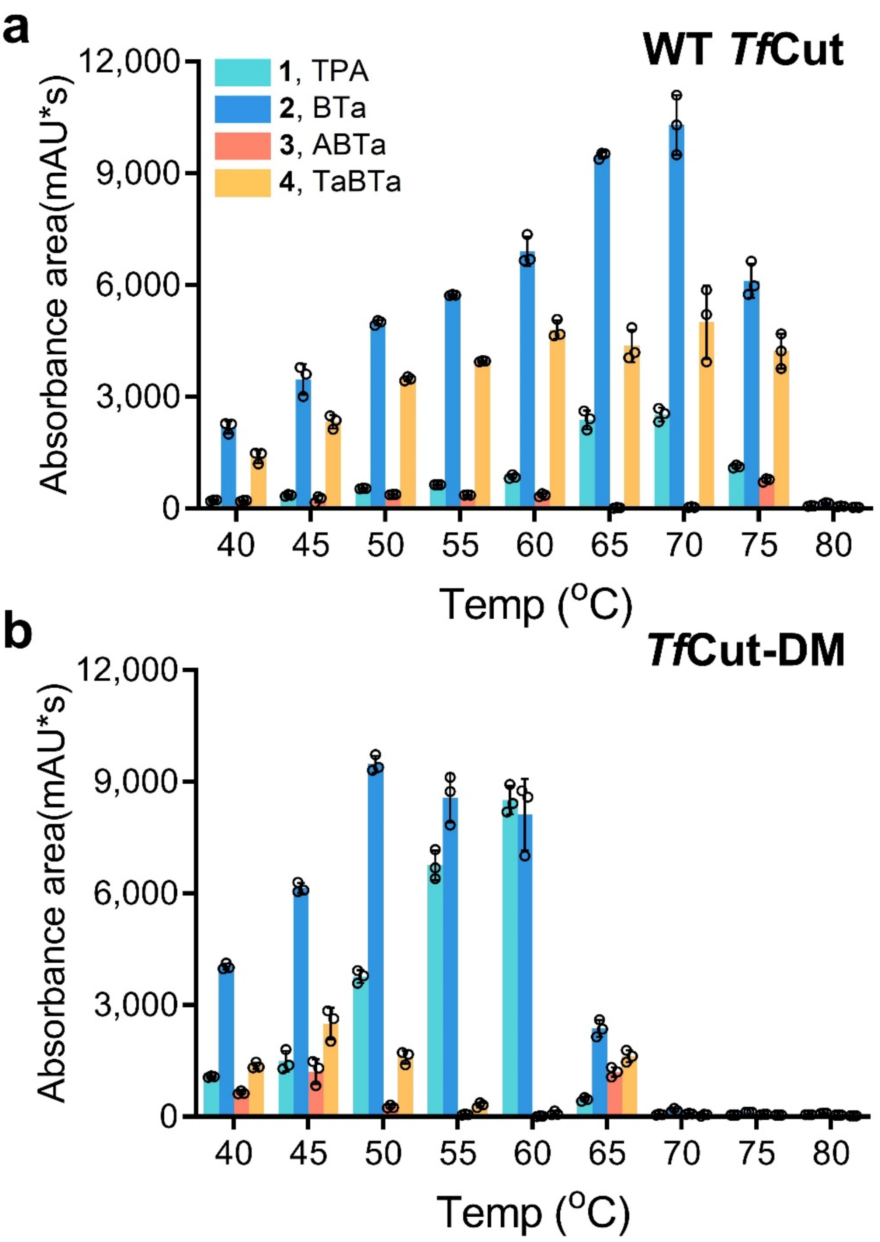

Supplementary Fig. 1. The temperature-dependent PBAT degradation rate of (**a**) WT *Tf*Cut and (**b**) *Tf*Cut-DM. The amounts of four intermediates in the reaction at 12 h were recorded by HPLC at the absorbance of 254 nm. Triplicate assay was conducted. Data are presented as mean values +/- standard deviation. The individual data points are shown as black circles. Source data are provided as a Source Data file.

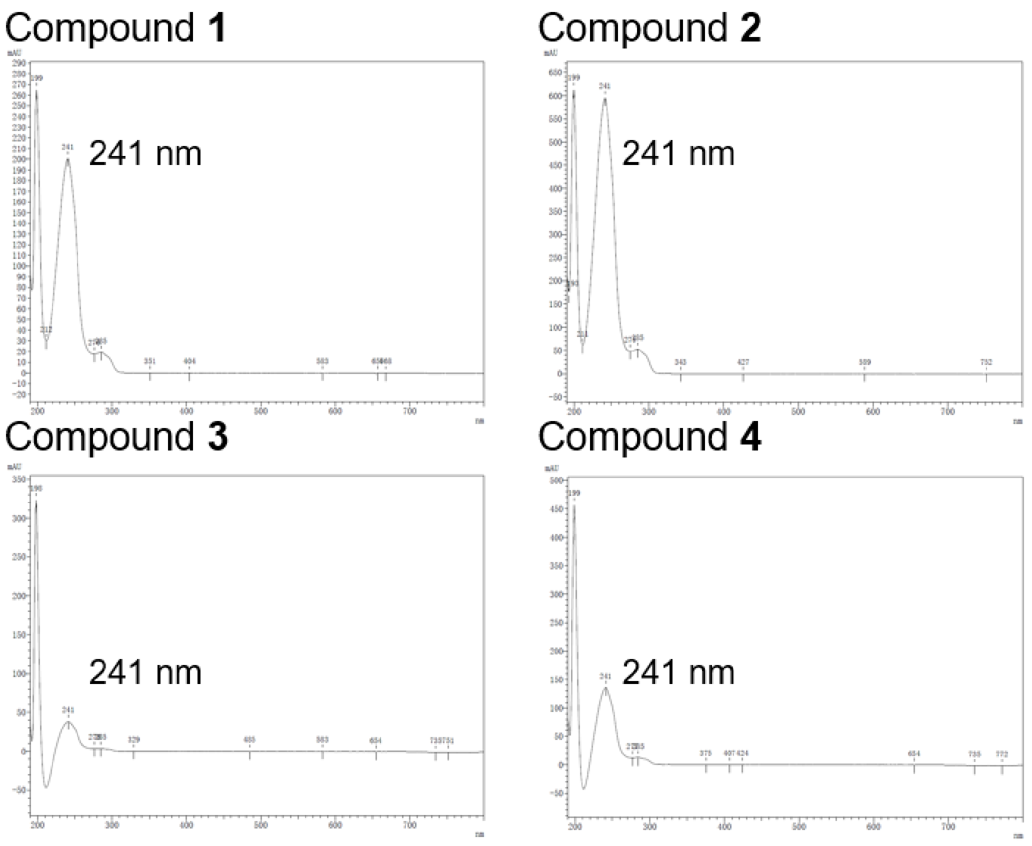

Supplementary Fig. 2. The four intermediates **1** – **4** show the maximal absorbance at 241 nm.

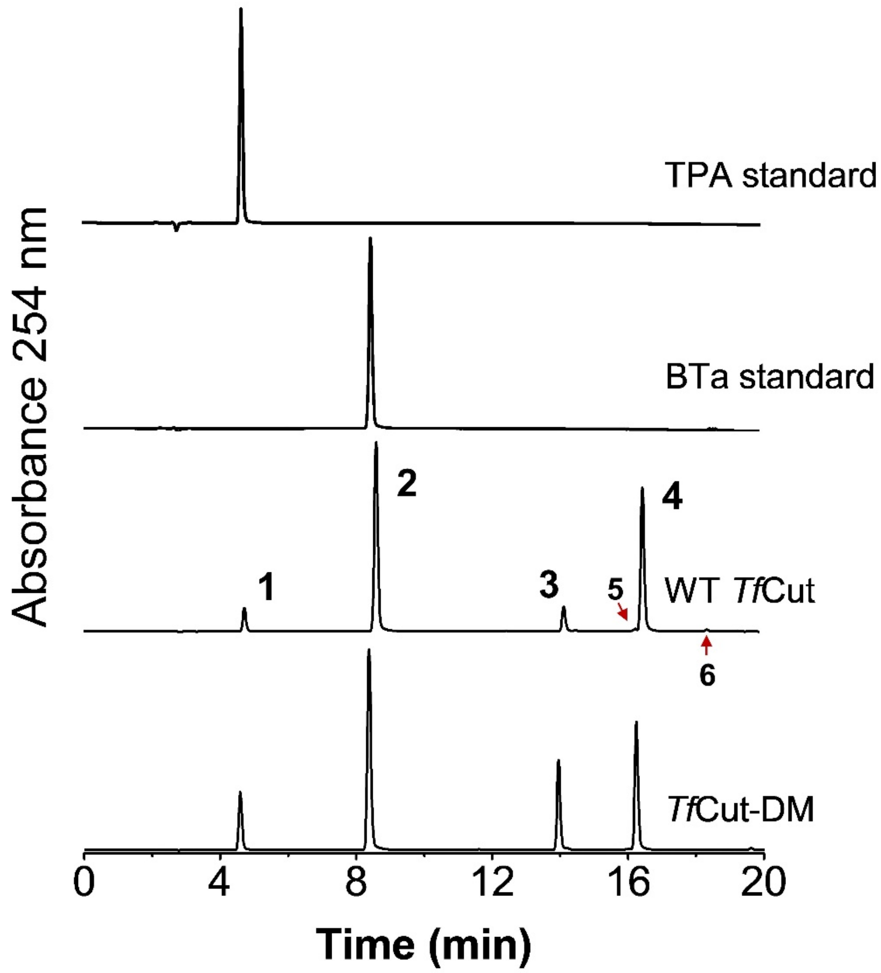

Supplementary Fig. 3. HPLC assay of PBAT hydrolyzed intermediates **1** - **6** and standard samples of TPA and BTa.

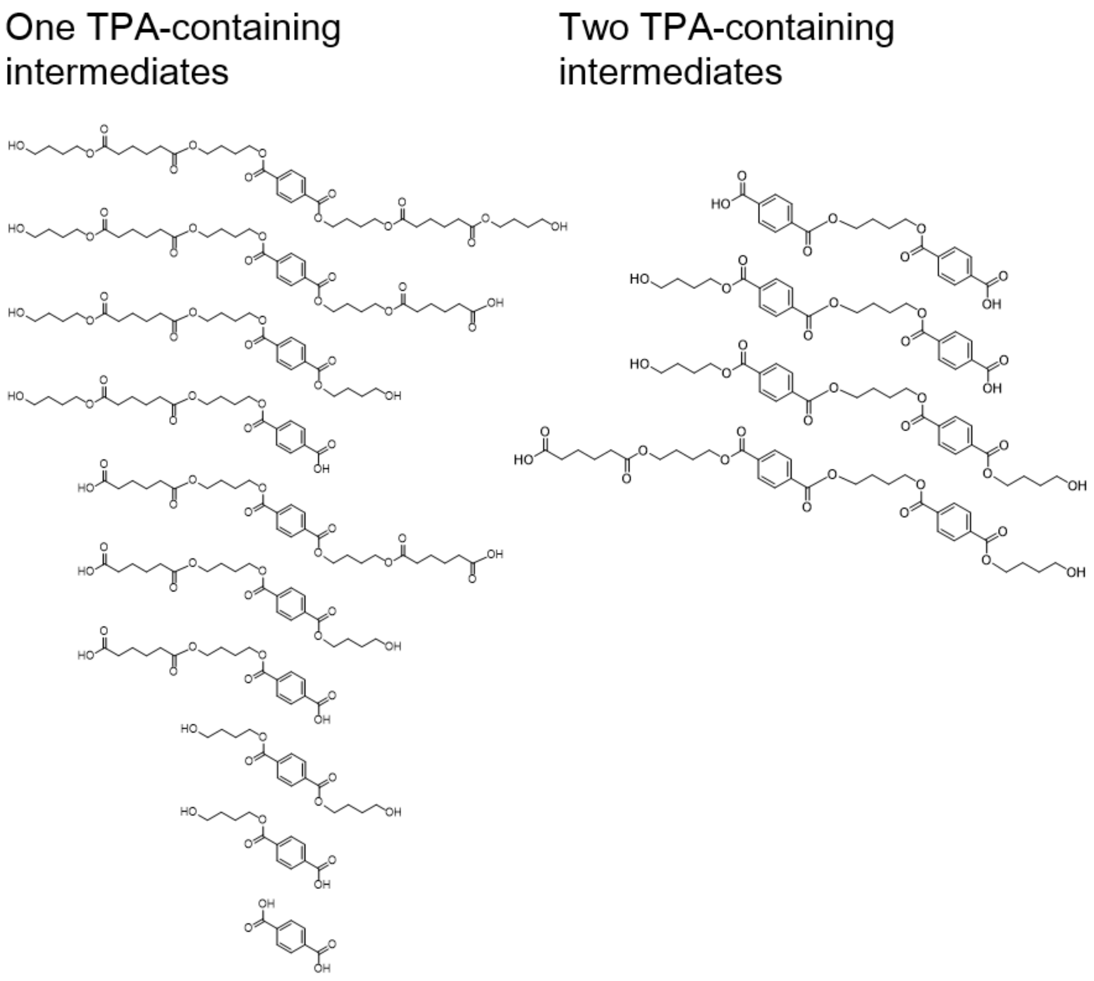

Supplementary Fig. 4. The speculation of hydrolyzed intermediates that contain one or two TPA during the PBAT-degradation.

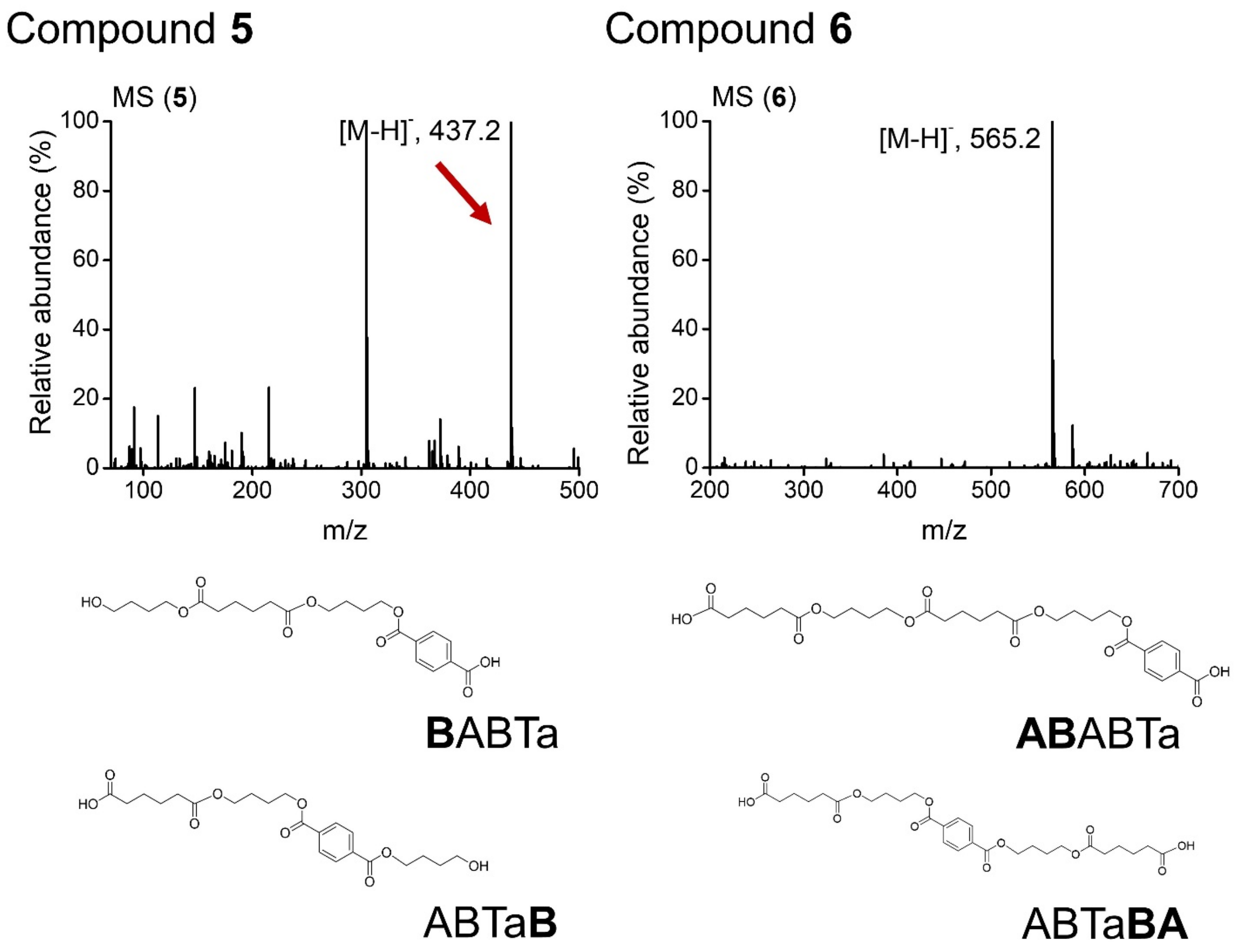

Supplementary Fig. 5. The mass spectrum of compound **5** and **6**, and their possible structures. The structure of **5** is BABTa or ABTaB. The structure of **6** is ABABTa or ABTaBA.

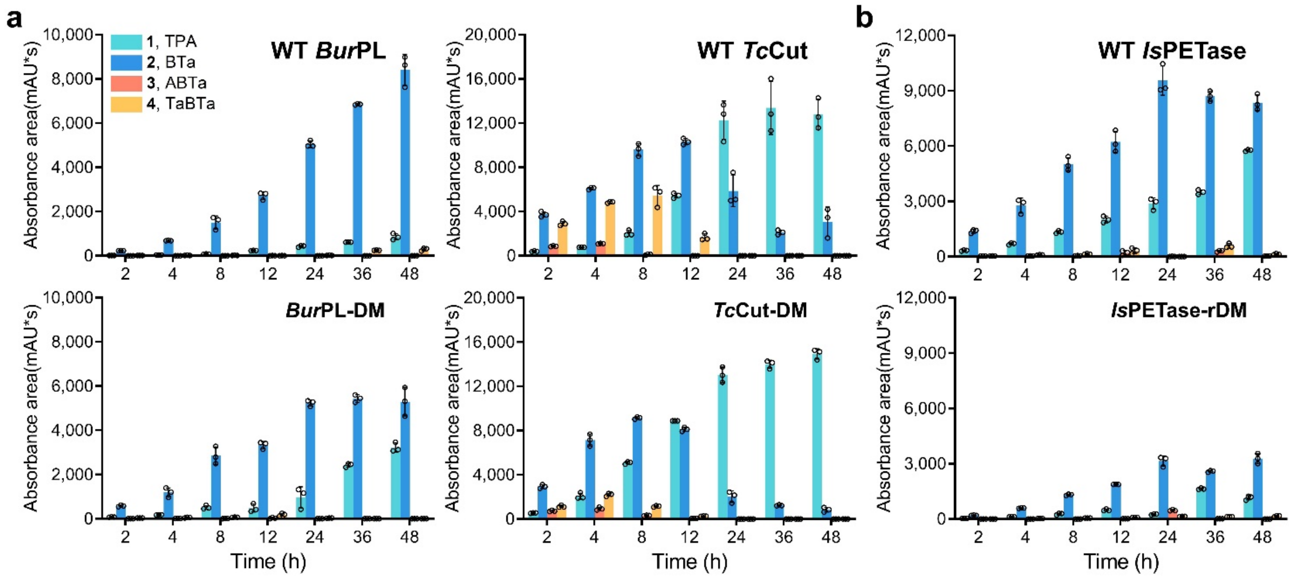

Supplementary Fig. 6. The time courses of PBAT-degradation mediated by **a**, WT and DM of *Bur*PL and *Tc*Cut; **b**, *Is*PETase and its rDM variant. The rDM indicates that the Ser/Ile duo was mutated to His/Phe. The operating temperatures of each enzymes are: WT *Bur*PL, 40 ºC; *Bur*PL-DM, 35 ºC; WT *Tc*Cut, 65 ºC; *Tc*Cut-DM, 60 ºC; WT *Is*PETase, 30 ºC; *Is*PETase-rDM, 30 ºC. Triplicate assay was conducted. Data are presented as mean values +/- standard deviation. The individual data points are shown as black circles. Source data are provided as a Source Data file.

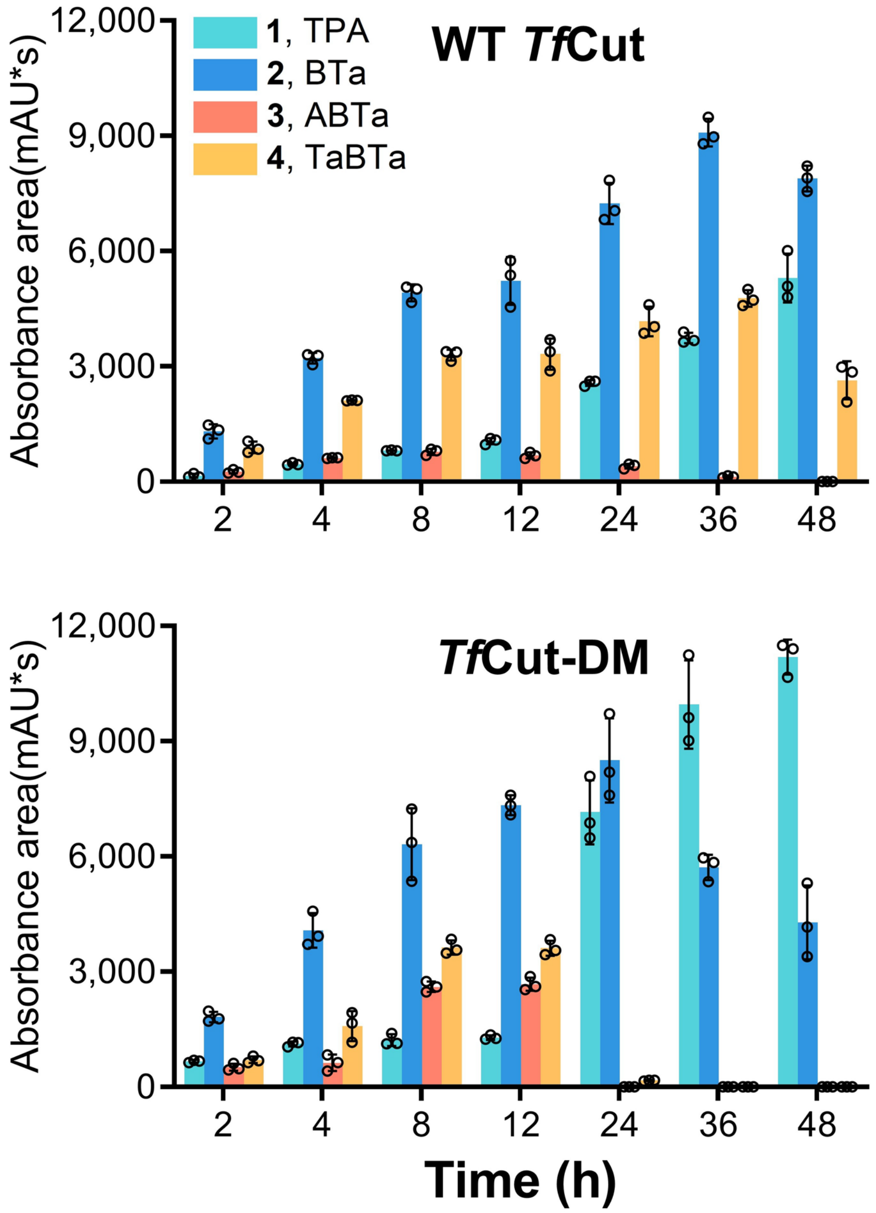

Supplementary Fig. 7. The time courses of WT *Tf*Cut (upper panel) and *Tf*Cut-DM (lower panel) mediated degradation of the UV-irradiated PBAT film. Triplicate assay was conducted. Data are presented as mean values +/- standard deviation. The individual data points are shown as black circles. Source data are provided as a Source Data file.

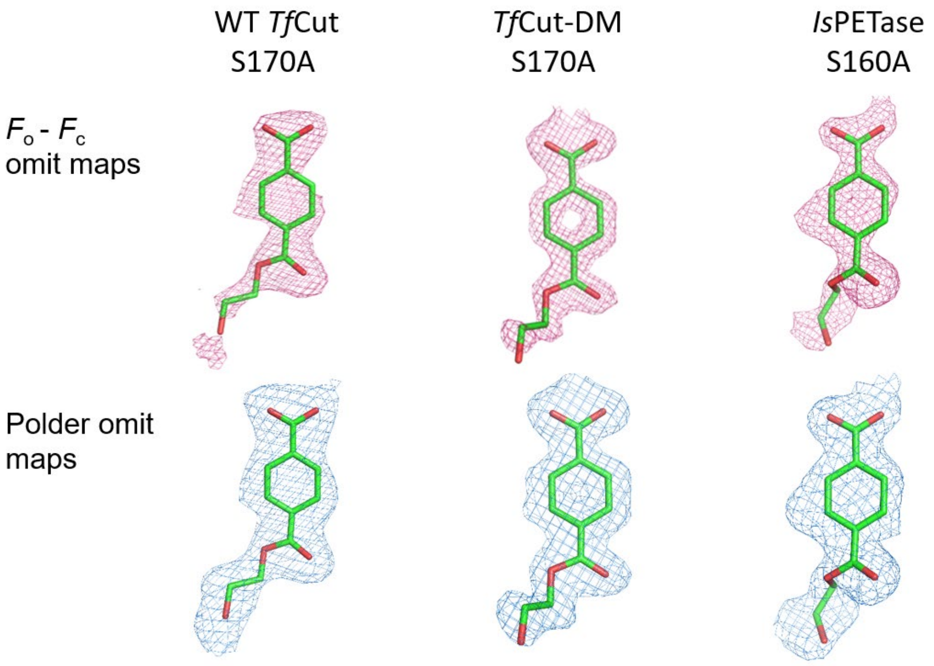

Supplementary Fig. 8. The F_o - F_c omit maps and polder omit maps of MHET in WT *Tf*Cut S170A, *Tf*Cut-DM S170A, and *Is*PETase S160A are contoured at 3.0 σ in pink and blue, respectively.

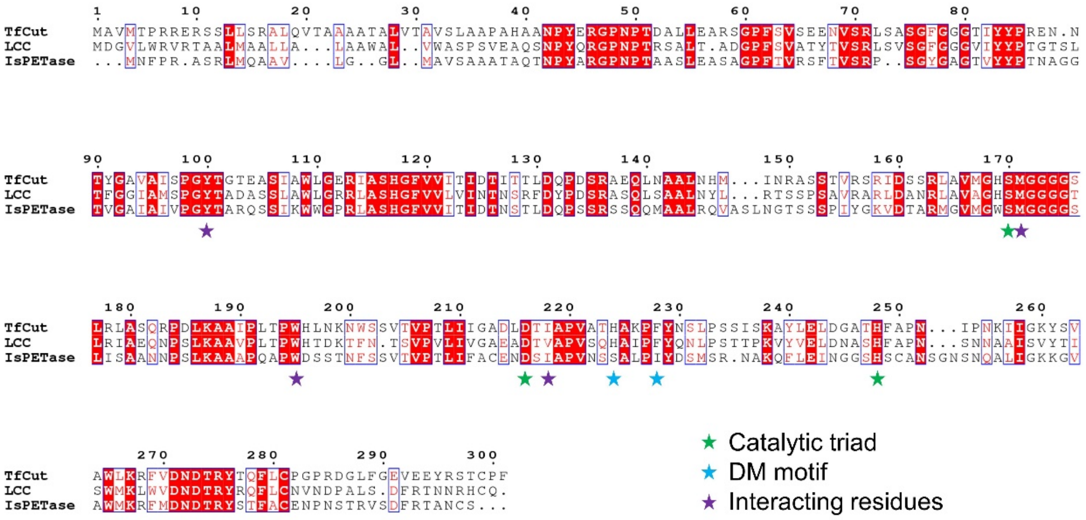

Supplementary Fig. 9. The sequence alignment of *Tf*Cut, *Is*PETase, and LCC. The residues of catalytic triad, DM, and interacting residues are labeled by green, blue, and purple stars respectively.

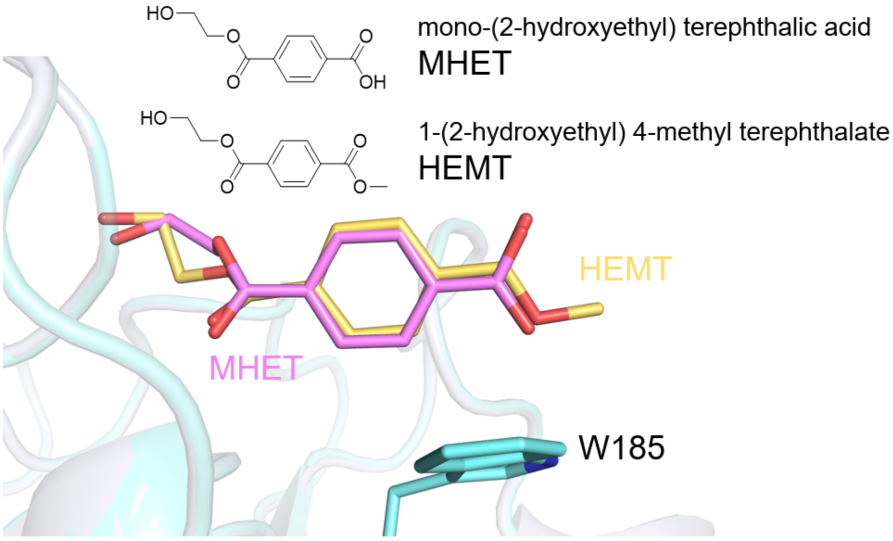

Supplementary Fig. 10. The comparison of MHET- (PDB ID: 7XTW) and HEMT-bound (PDB ID: 5XH3) *Is*PETase complex. The MHET and HEMT are shown as sticks in pink and yellow respectively.

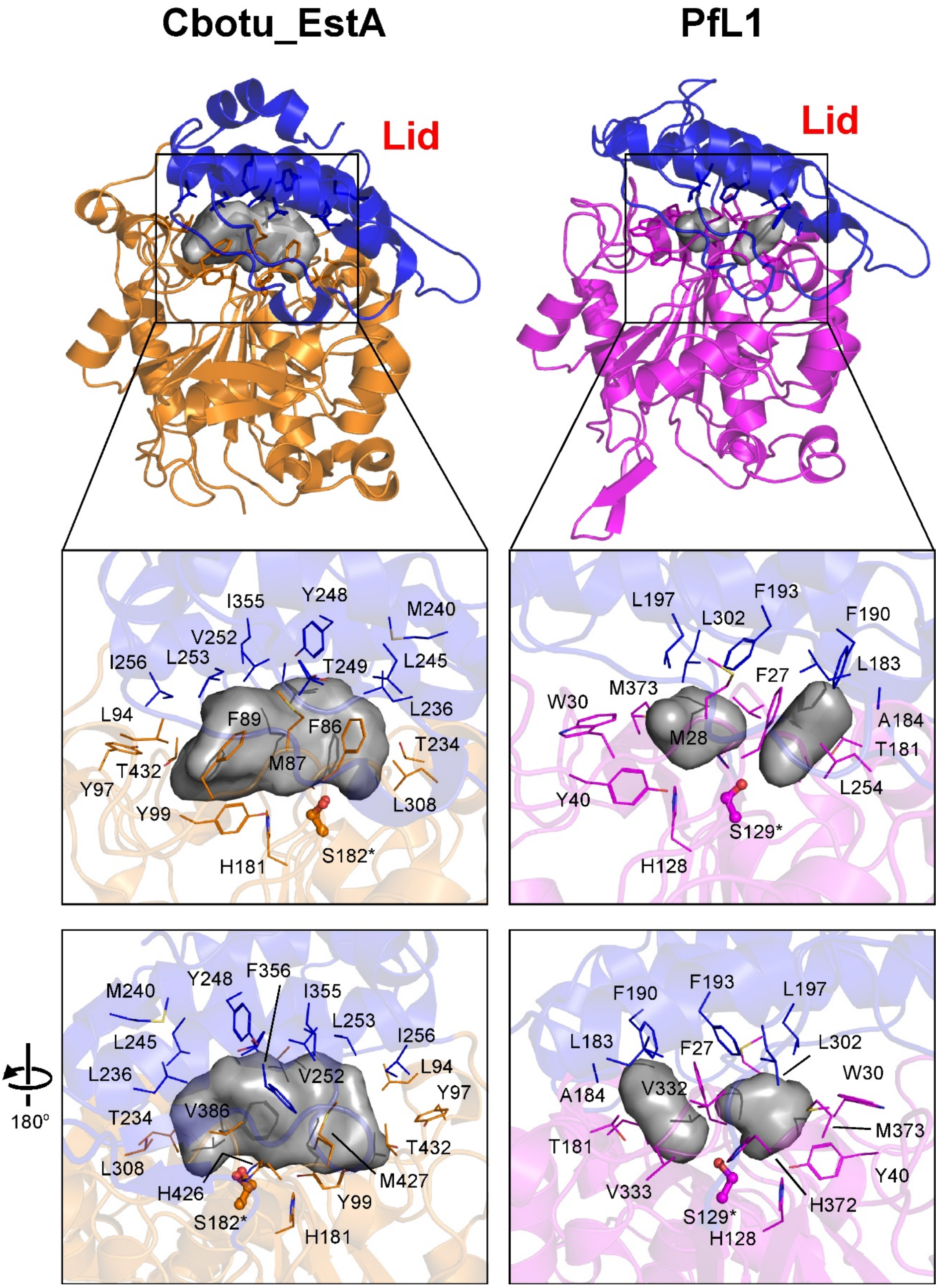

Supplementary Fig. 11. The active site pocket in the structures of Cbotu_EstA (PDB ID: 5AH1) and PfL1 (PDB ID: 5AH0). The cartoon models of both structures are shown on the top with respective lid domain colored in red. The putative substrate-binding pocket adjacent the catalytic residue are shown in the zoom-in views. The pocket-forming residues are shown in lines and the catalytic Ser are shown in stick and ball models.

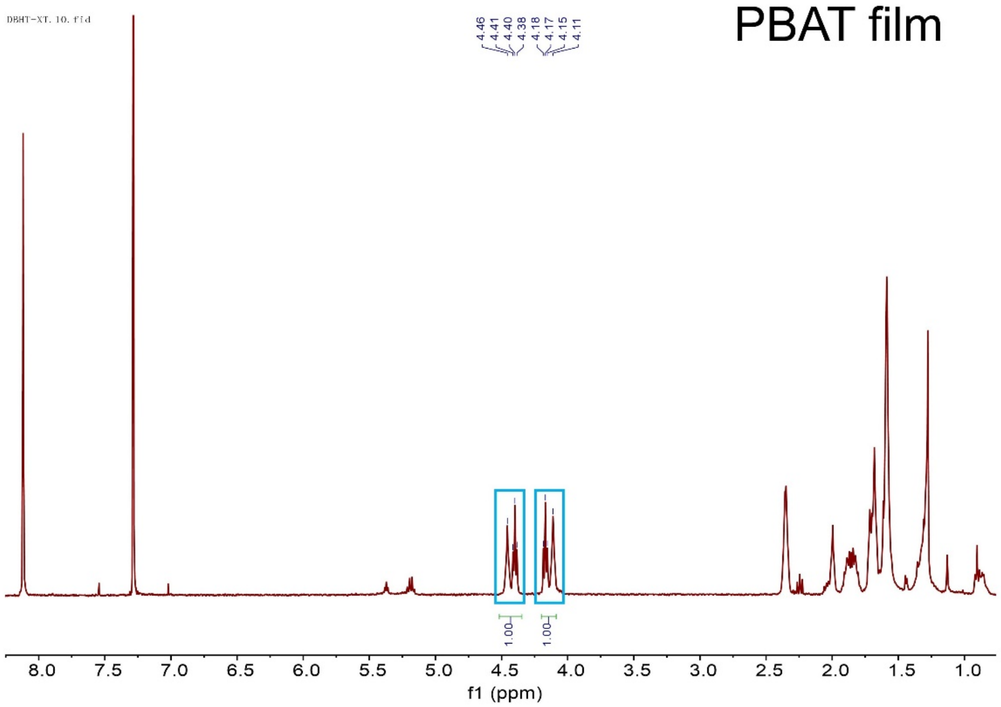

Supplementary Fig. 12. ^{1}H NMR ($CDCl_3$, 400 MHz) of the PBAT film used in this study. The ratio of Ada to TPA was calculated based on the peaks on 4.1 and 4.4 (boxed in blue) according to the method described by Hoe *et al.*[6].

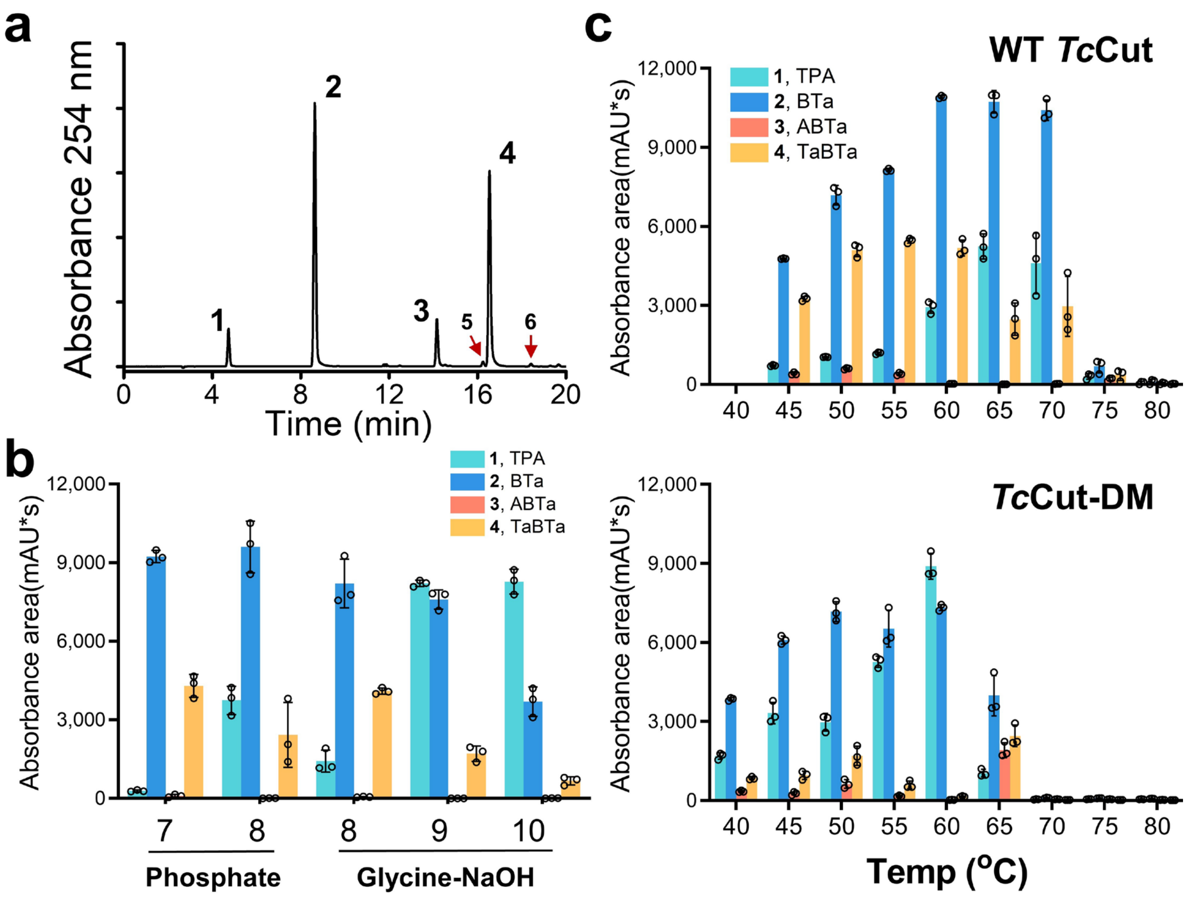

Supplementary Fig. 13. The characterization of *Tc*Cut in degrading PBAT. **a,** HPLC profile of *Tc*Cut-mediated PBAT degradation. The identities of compounds **1** - **6** are the same as those in *Tf*Cut-mediated PBAT hydrolytic products. **b**, Effect of pH on PBAT degradation of *Tc*Cut was estimated at 60 ºC for 12 h. **c**, The temperature-dependent PBAT degradation rates of WT *Tc*Cut (upper panel) and *Tc*Cut-DM (lower panel) were determined at 12 h by HPLC. Triplicate assay was conducted. Data are presented as mean values +/- standard deviation. The individual data points are shown as black circles. Source data are provided as a Source Data file

Supplementary References:

1. Biundo, A. *et al.* Characterization of a poly(butylene adipate-co-terephthalate)-hydrolyzing lipase from *Pelosinus fermentans*. *Appl. Microbiol. Biotechnol.* **100**, 1753-1764 (2016).
2. Wallace, P. W. *et al.* PpEst is a novel PBAT degrading polyesterase identified by proteomic screening of *Pseudomonas pseudoalcaligenes*. *Appl. Microbiol. Biotechnol.* **101**, 2291-2303 (2017).
3. Perz, V. *et al.* Hydrolysis of synthetic polyesters by *Clostridium botulinum* esterases. *Biotechnol. Bioeng.* **113**, 1024-1034 (2016).
4. Engh, R. A. & Huber, R. Accurate bond and angle parameters for X-ray protein structure refinement. *Acta Crystallogr. Section A* **47**, 392-400 (1991).
5. Chen, V. B. *et al.* MolProbity: all-atom structure validation for macromolecular crystallography. *Acta Crystallogr. D Biol. Crystallogr.* **66**, 12-21 (2010).
6. De Hoe, G. X. *et al.* Photochemical Transformation of Poly(butylene adipate- co-terephthalate) and Its Effects on Enzymatic Hydrolyzability. *Environ. Sci. Technol.* **53**, 2472-2481 (2019).
